# Tool Condition Monitoring for High-Performance Machining Systems—A Review

**DOI:** 10.3390/s22062206

**Published:** 2022-03-12

**Authors:** Ayman Mohamed, Mahmoud Hassan, Rachid M’Saoubi, Helmi Attia

**Affiliations:** 1 Department of Mechanical Engineering, McGill University, Montreal, QC H3A 0C3, Canada; ayman.mohamed2@mail.mcgill.ca (A.M.); helmi.attia@mcgill.ca (H.A.); 2 Advanced Material Removal Processes, Aerospace Manufacturing Technologies Center (AMTC), National Research Council Canada, Ottawa, ON K1A 0R6, Canada; 3 R&D Material and Technology Development, Seco Tools AB, SE-73782 Fagersta, Sweden; rachid.msaoubi@secotools.com

**Keywords:** tool condition monitoring, machine learning, sensor fusion, milling process, signal processing, feature extraction

## Abstract

In the era of the “Industry 4.0” revolution, self-adjusting and unmanned machining systems have gained considerable interest in high-value manufacturing industries to cope with the growing demand for high productivity, standardized part quality, and reduced cost. Tool condition monitoring (TCM) systems pave the way for automated machining through monitoring the state of the cutting tool, including the occurrences of wear, cracks, chipping, and breakage, with the aim of improving the efficiency and economics of the machining process. This article reviews the state-of-the-art TCM system components, namely, means of sensing, data acquisition, signal conditioning and processing, and monitoring models, found in the recent open literature. Special attention is given to analyzing the advantages and limitations of current practices in developing wireless tool-embedded sensor nodes, which enable seamless implementation and Industrial Internet of Things (IIOT) readiness of TCM systems. Additionally, a comprehensive review of the selection of dimensionality reduction techniques is provided due to the lack of clear recommendations and shortcomings of various techniques developed in the literature. Recent attempts for TCM systems’ generalization and enhancement are discussed, along with recommendations for possible future research avenues to improve TCM systems accuracy, reliability, functionality, and integration.

## 1. Introduction

The benefits of advances in digital technologies, along with the development of the Industrial Internet of Things (IIoT) have expanded at a rapid rate over the last two decades. This is due to the development of smart sensing technologies and data storage capacities that has led to the ‘Industry 4.0’ revolution, where advanced manufacturing techniques are combined with IIoT systems to drive further intelligent action back in the physical world, motivating unmanned manufacturing. This drives competitive industrial advantages in terms of reducing cost, increasing productivity, improving quality, and preventing damage to machined parts during processing. An intelligent tool condition monitoring (TCM) system is a building block in this framework to achieve such automated machining systems. It provides a digitalized feedback estimation of the tool condition based on analytical or sensor-based models to safeguard the machined part, and to enable process optimization and quality control in real-time. Therefore, tremendous efforts have been exerted towards developing new methods and implementing innovative technologies to improve the performance of TCM systems and to introduce solutions to the challenges facing manufacturers.

**Figure 1:**
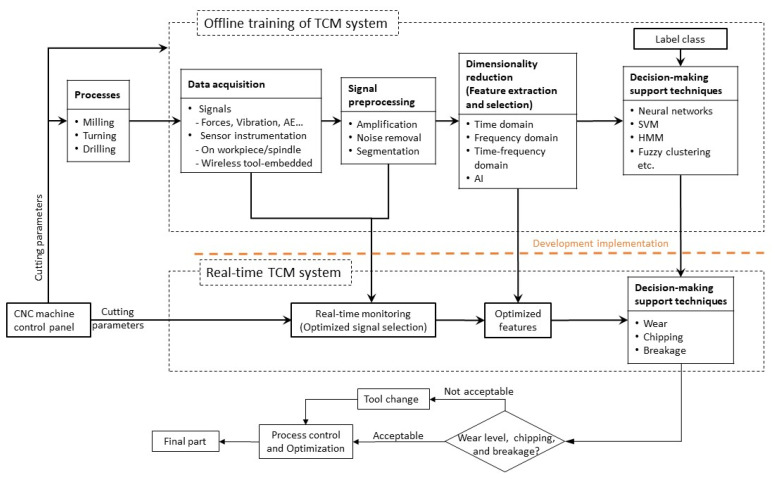
Tool condition monitoring in machining processes.

Tool condition monitoring systems can be applied offline, online, or in real-time. An offline TCM system entails interrupting the machining process and examining the tool health state using inspection equipment such as an optical microscope at unregulated periods [1]. In online TCM systems, the machining process is not interrupted, but the monitored parameters are measured and related to the tool health state at regulated intervals without constraints on either the acquisition time intervals or the time needed to process the captured data to take a corrective action [2]. Real-time TCM systems continuously acquire process data at fully regulated time intervals without interrupting the machining process, but with limited latency. This enables taking corrective action to avoid cutting tool failure and workpiece damage, and can be employed in an adaptive control (AC) system to execute dynamic tool compensation to improve the accuracy and economics of the machining process [3,4]. However, it allows a short time span for acquiring and processing the monitored signals and predicting the tool health state, which requires algorithms with low computational cost [2]. A real-time intelligent TCM system commonly consists of four stages, as illustrated in Figure 1, namely, signal acquisition, signal pre-processing, features construction and selection, and tool health model. The system is trained offline at first to optimize the selected sensors and the extracted indicative features. Later, the selected sensors and features are used during the real-time system implementation to define the level of tool failure. Corrective actions such as cutting feedrate optimization or tool change can be executed based on the tool health state. Acquired data can be categorized, according to their measurement techniques, into direct and indirect techniques. The utilization of machine vision [5,6] and optical microscopy [7] to directly measure the amount of tool wear are generally reliable. Despite this, they are neither efficient, cost-effective, nor feasible, compared to the indirect methods [8] owing to the harsh machining environment and the required process interruptions to identify the tool health state [8]. Furthermore, direct techniques are unable to identify any unexpected cutting tool damage (chipping and/or breakage) during the tool/workpiece engagement. Thus, indirect measurement techniques have been developed for real-time monitoring to take immediate action when it is necessary. In these techniques, auxiliary measured variables, such as cutting forces, torque, vibration, acoustic emission, and power signals are correlated to the tool health state. Despite the applicability and cost-effectiveness of the indirect measurement techniques, they are less accurate and heavily dependent on process parameters, and their signals are noisy, and affected by the machining environment. Hence, advanced signal processing techniques are needed to overcome these challenges by extracting indicative features that accurately represent the tool health state from the acquired signals. This increases the reliability and robustness of the TCM, which would help in avoiding false alarms and poor performance of the process control algorithms.

Many techniques have been presented to model the tool health states in TCM systems, which can be classified into two categories: physics-based, and data-driven models. Physics-based models usually simplify the cutting processes using a semi-empirical law or mechanistic model [9]. They can be extrapolated for usage in untested machining situations and provide insight into the internal functioning of the machining process. Due to the complex nonlinear nature of the cutting process, several factors impacting the cutting process are neglected, e.g., the cutting temperature and the lubrication conditions, which limit the prediction accuracy [2]. As a result, physics-based models developed in the literature, such as the Taylor model [10], the generic tool wear model [11], and others [12,13], cannot be used for accurate real-time TCM. Hence, data-driven models have received much attention for tool wear modeling in which the monitored signals are correlated to the tool health state through conventional and deep machine learning techniques, which are subsets of artificial intelligence (AI) techniques. As part of the Industry 4.0 revolution, the rapid advancements in computing systems have facilitated the day-by-day utilization of such models because of the massive computation required as the number of input features in the model grows.

High-performance machining involves overall process optimization through fully utilizing the machine capabilities to minimize production costs, boost productivity, meet pre-defined component quality characteristics, and maximize tool life. It encompasses continuous optimization of the cutting speed, feedrate, and strategies, either offline or online. Therefore, a real-time autonomous TCM system with high degree of generalization and robustness is essential to accommodate this continuous change with minimum calibration efforts and without process disturbance. In this paper, more than 200 recent publications have been analyzed to furnish the knowledge of recent advancements in TCM. Furthermore, research gaps and limitations of the recently developed approaches are highlighted. Despite the numerous literature reviews on the development of TCM systems [2,14,15,16,17,18], there is a lack of discussion and analysis on dimensionality reduction techniques that represent a crucial stage in the identification of features that are highly sensitive to the tool health state only and independent of change in process parameters. Additionally, to the best of the authors’ knowledge, a review of the development of wireless tool-embedded TCM systems has not been presented in the literature yet. Therefore, this work aims to provide an in-depth analysis and discussion of various designs of wireless TCM tool-embedded sensor nodes found in the literature.

The article is organized as follows: Section 2 discusses the challenges and benefits of the most common indirect sensors used for TCM. In addition, it analyzes the recent trials to design a universal wireless sensor node with a focus on tool-embedded sensors, wireless transmission protocols, and power management techniques. The needed signal pre-processing techniques and the subsequent signal processing algorithms to generate informative features for the decision-making stage are evaluated in Section 3 and Section 4, respectively. Section 5 and Section 6 presents the literature related to implementing cutting tool wear monitoring and recent advances in detecting and preventing tool chipping/breakage, respectively, followed by a conclusion for main research gaps and possible opportunities to develop an accurate, robust, and generalized TCM system that meets the requirements of the industry.

## 2. Sensing and Data Acquisition

As indicated earlier, indirect methods are preferred, as real-time tool health indicators, by establishing a correlation between the measured process parameters and the tool health state. Commonly monitored indirect parameters in TCM systems include cutting forces [19], vibrations [20], acoustic emissions AE [21], and spindle motor feedback signals [22]. In addition, other parameters such as cutting-edge temperature [23] and the spindle rotational speed can be monitored to detect the tool health state, but with lower feasibility in industrial applications. The conventional approach is to mount desired sensors on the spindle or workpiece. Recently, an approach was proposed to increase the reliability and universality of TCM systems by mounting sensors on the tool holder to have a universal wireless sensors node, which comes with its own challenges and benefits, as will be discussed in Section 2.2.

### 2.1. Conventional Means of Sensing

#### 2.1.1. Cutting Force Signal

Due to its high sensitivity to tool conditions, the cutting force signal is the most reliable and stable variable in machining operations, which makes it the most commonly utilized signal to detect tool wear [24,25]. As the machining process progresses, the cutting tool loses its sharpness and becomes dull, leading to a rise in the friction force between the tool and workpiece and the cutting force needed to remove chips from the workpiece material under the same cutting conditions [26]. The increase in cutting forces can also be attributed to other factors, including the cutting conditions, the material of the cutting tool, and the material of the workpiece. Therefore, a normalizing approach is necessary to accentuate the tool wear effect on the acquired signals and mask out all other factors [27]. For difficult-to-cut material such as Ti6Al4V, the cutting force might not increase at a certain limit due to the thermal softening mechanism that competes with the strain hardening effect [28]. This can create a false alarm by the TCM system when it operates under varying cutting conditions. Cutting forces can also be utilized in chatter detection if the used sensor bandwidth can cover the chatter frequencies [29]. The table dynamometer is a very popular sensor for force measurements in indirect TCM developments in academia due to its high sensitivity and reliability as it is placed under the machined part, resulting in detecting small load changes [16]. However, it is impractical to use in industrial facilities owing to its high cost and the need to protect it from overloads [30]. Moreover, the table dynamometer limits the size of the machined part and reduces the machining system rigidity [31,32]. To overcome most of these weaknesses, integrating the force sensors into the tool holder has been suggested as a way to increase the practicality of such a technique for industrial applications but at even higher cost [33].

#### 2.1.2. Vibration Signal

The cutting tool vibrations are measured by piezoelectric or micro-electromechanical system MEMS accelerometers to predict the tool edge wear and the surface roughness of the machined surface, among others [34,35]. Sharp cutting tools create modest amount of vibrations that rise as the tool condition deteriorates [36]. Tool vibrations result in undesired displacements of the cutting tool, which have a strong relationship with the roughness and waviness of the machined surface [37]. Vibrations generated during metal cutting may be classified into cutting-dependent and cutting-independent vibrations [17]. Cutting-dependent vibrations demonstrate the characteristics of the cutting process, such as interrupted cutting, while cutting-independent vibrations include forced vibrations caused by machine components, such as unbalanced rotating parts. It is highly important to process the signal to distinguish between both types of vibrations for an accurate representation of tool wear [25]. A vibration sensor is easy to install and less expensive compared to other sensors, such as AE sensors and dynamometers. However, the signals are notoriously difficult to filter, making them prone to delivering inaccurate information [36]. Moreover, the transmission path of the signal from the vibration source to the location of the vibration sensor, and the cutting fluid have direct impact on the vibration signal.

#### 2.1.3. Acoustic Emission Signal

Acoustic emission AE sensors are used to capture the radiation of the acoustic waves released from irreversible processes within a material, such as wear, chipping, and breakage of the cutting tool, chip formation, and thermal reaction. Since the frequency bandwidth of the AE waves (100 kHz–1 MHz) is higher than that of machine vibrations and ambient noises (1 Hz–10 kHz), the AE signal is widely considered as one of the most effective methods for detecting tool wear and breakage [38]. In addition, the AE signal can anticipate incoming events by monitoring acoustic waves generated during the unstable crack propagation in the prefailure stage, offering the chance to take precautions for unexpected and undesirable events [39,40]. In this way, the AE technique may be utilized as an early warning system, particularly for preventing failures, which can be beneficial in practice for lowering production cost [41]. Depending on the source of the signal, AE signals in the cutting process are composed of both continuous and transient signals. Shearing in the primary shear zone and wear on the tool flank face create continuous signals, whereas transient or burst AE signals are generated by tool engagement and disengagement with the workpiece, tool fracture, or chip breakage, among others [42]. In the open literature, reported data on the effectiveness of the AE sensor in monitoring the tool condition are contradictory when it comes to the two suggested locations for mounting the AE sensor; either on the spindle or on the workpiece. However, it produces more reliable signals when mounted on the spindle due to the closeness to the signal source at the cutting zone and the short signal transmission path [43,44]. While AE sensors are relatively inexpensive and easy to integrate on the machine, they must be calibrated properly as the signal transmission path, the reflective surfaces between the cutting zone and the sensor, and the machine condition, can influence the quality of the AE signal [17].

#### 2.1.4. Motor Current Signal

The primary source of energy in cutting operations is the spindle motor current, which is linked to changes in the cutting zone, including the tool health state. With the progression in the tool edge wear, the cutting forces increase, which increases the drawn current [45]. The inertia of the motor rotor acts as a low-pass filter, which limits the bandwidth of the detected signal and the detection of the high-frequency change in cutting forces [38]. Therefore, if the motor frequency is lower than the tool-pass frequency, the captured signal may lose some information [46]. Nonetheless, modern computer numerical control (CNC) machines are equipped with 400 Hz two-pole induction motors, allowing for frequency ranges of up to 24,000 rpm [47,48]. Compared to other sensors, the use of current sensors in the TCM systems reported in the literature is minimal [16]. However, it is the main signal used by commercial TCM systems, where dynamic threshold approaches are commonly used to define the tool condition [49]. This threshold varies according to the cutting conditions and the workpiece material. Although motor current sensors are economical and easy to install without interfering with the cutting zone [50], the signal is not sensitive to the cutting force fluctuations at high spindle speeds and is influenced by the machine condition and the viscous damping of the feed system [51,52].

#### 2.1.5. Temperature Signal

Despite being able to monitor the tool wear level, temperature sensors utilization in real-time TCM systems is rare owing to the high thermal inertia, the low response of the embedded conventional thermocouples [23], and the difficulty of embedding the temperature sensor in a rotating tool close to the cutting edge, e.g., in milling processes. Utilizing a thermal imaging camera is another approach that can be used to measure the concentrated heat at the cutting zone of Ti6Al4V [19]. However, such a technique is not appropriate in the harsh machining environment. To overcome the low response of the conventional thermocouple, He et al. [53] utilized a temperature signal from a thin-film thermocouple embedded into a cutter in turning operations to monitor the tool wear. Under varying cutting conditions, the authors have reported high predictions levels, which highlights the robustness of such signals to improve the wear predictions. In the milling operations of hard-to-cut materials, monitoring the cutting zone temperature is important due to the varying wear mechanisms that are triggered by the high cyclic thermal loading [54].

#### 2.1.6. Spindle Rotational Speed Signal

The repetitive shocks and friction between the cutting tool and the workpiece are the primary source of the spindle speed fluctuations [55]. Very few studies relied on this signal to detect chatter and to monitor tool wear and breakage [55,56,57], by using the spindle motor encoder to monitor the instantaneous spindle speed but with a low resolution of less than 150 Hz. A higher resolution can be achieved by embedding a gyroscope sensor on the tool holder [58]. Along with the cutting torque signal, an accurate measurement of the cutting power can be made in real-time, which can provide instantaneous feedback about the tool health state and the cutting process for AC systems compared to the motor current.

#### 2.1.7. Multi-Signal Approach

A multi-sensor approach, in which the TCM system considers monitoring several process and machine parameters, is preferable to increase the TCM accuracy and reliability [59]. This has been reflected in the progressive growth of the number of studies focusing on equipping the TCM systems with multi sensors for milling operations [1,60,61,62,63,64]. Apart from the externally mounted sensors, modern CNC machines allow real-time data acquisition from their internal sensors and control system, such as spindle speed, feedrate, and spindle motor power feedback that can be used in TCM systems [65]. Despite their high reliability, their utilization in TCM systems is limited. This is due to the low sampling frequency that is commonly < 250 Hz, which does not cover the machining frequency bandwidth in high-performance machining applications [66]. The multi-sensor approach increases the system robustness, spatial and temporal information resolution, and the ability to cover a wider range of phenomenon frequencies [28,67]. The number of sensors utilized in TCM systems must not be excessive to avoid the associated increase in the expenses of manufacture and maintenance, the interference with the machining process, and the redundant data that might degrade the detection accuracy [25,66]. Therefore, an in-depth investigation to define and optimize the essential sensors and their features based on the monitored process is required [68]. One of the few studies that investigated various combinations of signals, including forces, vibrations, AE, sound, and current for tool wear monitoring was carried out by Ghosh et al. [69]. Based on performance and economic feasibility, the study suggested the current and sound-based TCM system for the general machining industry, and the current and force-based TCM system for the high-value machining industry. The work conducted by Duo et al. [66] on the predictive capacity of a group of time domain features for various internal and external signals in drilling operations concluded that the externally measured feed force and the internally measured spindle torque are the most sensitive signals to monitor the tool health state. Based on the surveyed literature, previous works lack such investigation, and sensors selection is always determined by scientists based on experience, ease of integration, and availability, among others.

**Figure 2:**
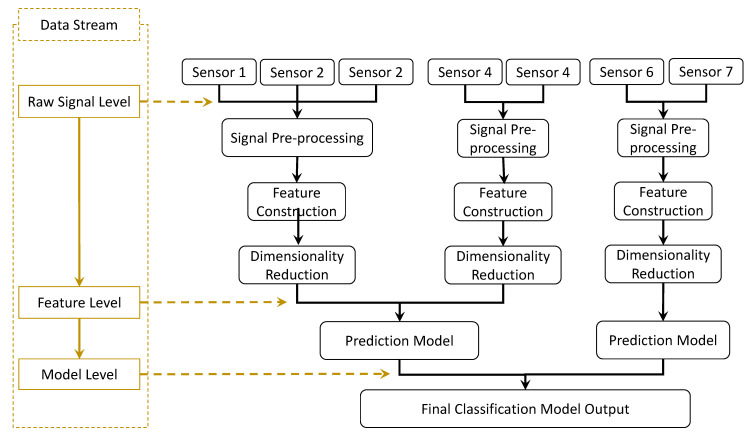
Arbitrary tree for data fusion levels.

When designing a multi-signal TCM system, the acquired data are fused at either the raw signal, feature, or model levels [70], as shown in Figure 2. Fusing the acquired signals at the feature level is used in most TCM research, where different features from multiple signals are selected and employed in the tool wear prediction model [25,64]. By fusing the data at the model level, two or more tool wear classifiers are merged to generate a more confident decision using a voting function [71]. Few studies have fused the raw data acquired from multiple sensors, e.g., the study carried out by Kuljanic et al. for the face milling operations [72], in which the torque and cutting force signals are divided to introduce a new variable called the torque-force distance indicator (TFD). The TFD showed a strong correlation to the tool wear and was independent of the cutting parameters compared to the normalized cutting forces. Investigating the fusion of acquired data at different levels might explore new variables and features that are only highly sensitive to the tool health state and independent of the cutting parameters.

### 2.2. Universal Sensor Node Approach

**Figure 3:**
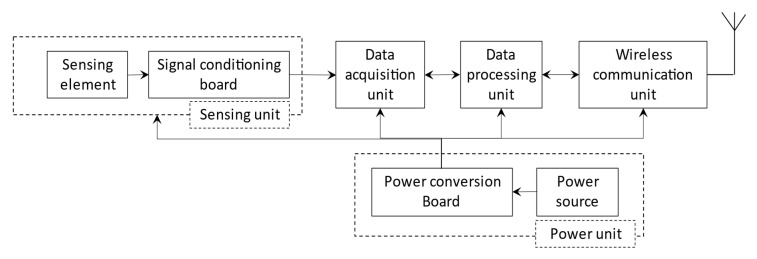
Wireless sensor node structure.

**Figure 4:**
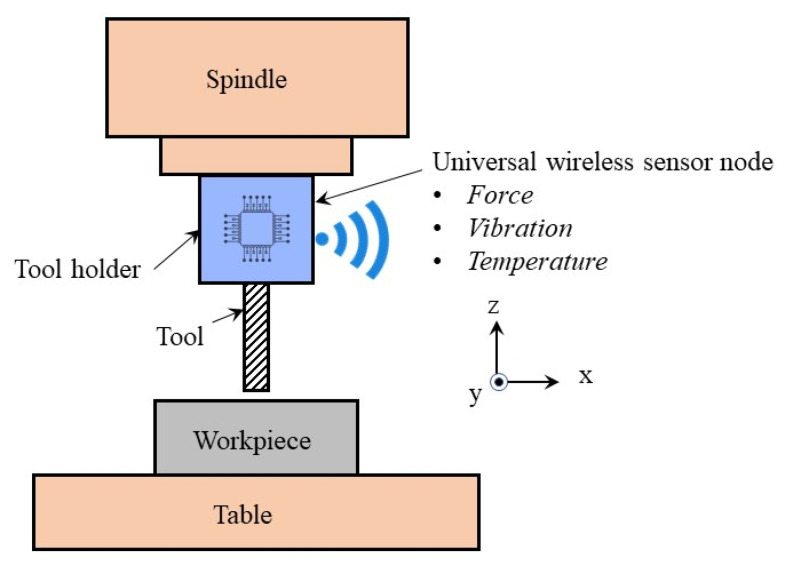
Universal wireless tool-embedded sensor node in the milling process.

Wireless TCM systems provide high implementation flexibility, accessibility, and connectivity compared to wired sensor-based systems. Wireless sensor networks (WSN) provide an efficient and effective solution for TCM systems and other condition monitoring applications [68,73,74,75]. Along with the obvious benefits of cheap installation and operating cost, WSN also offers low power consumption, and remarkable universality when used with different machine setups [76,77]. WSN is an ad hoc local area network that consists of one or several wireless sensor nodes. Generally, a wireless sensor node is composed of a mean of sensing, data acquisition, data processing, wireless communication, and power units, as shown in Figure 3 [75]. In machining processes, a sensor node can be mounted on the tool holder, as shown in Figure 4, close to the source of the signal at the cutting zone to provide a better quality of the detected signal than the conventional sensor mounting approach on the machine spindle or worktable [78]. This requires the sensor node to be able to operate in a harsh and confined space close to the signal source with minimum intervention in the workspace for successful integration into TCM systems [79]. It should also provide the required high-resolution data sampling for accurate and reliable tool state health decisions. Additionally, such a system should have the potential to integrate multiple sensors to increase the TCM system accuracy [79]. The universal sensor node concept is still in the proof-of-concept stage and has been utilized in very few studies to estimate either the tool wear or the surface roughness of the machined part [80,81]. The following subsections discuss the design considerations and challenges in developing a robust universal sensor node for TCM systems.

#### 2.2.1. Sensory Integration

Mounting the sensing unit at a far physical distance from the signal source weakens the acquired data and introduces attenuation and noise to measurements by other components of the machine, such as spindle bearings and collet interfaces [78,82]. For instance, vibration signals measured at the tool holder and the machine spindle were compared to reveal the deterioration in the signal quality caused by the damping effects experienced in the spindle assembly [43,83,84]. Integrating accelerometers within the TCM sensor node is relatively a straightforward task. Researchers usually pick a commercial piezoelectric senor [84] or MEMS sensor [78] with appropriate bandwidth and mount it on the rotational axis of the tool holder. In terms of cost, weight, and volume, MEMS sensors are superior to piezoelectric sensors, but their signal-to-noise ratio and bandwidth are limited [85].

More reliable AE signals can be gained by mounting the AE sensor on the tool holder close to the signal source, which avoids multiple acoustic wave reflections and shortens the transmission path [86]. To the best of the authors’ knowledge, no study has successfully embedded the AE sensor into a wireless sensor node in a rotating tool. One unavoidable obstacle to the practical application of AE in rotating machine fault detection is the frequency range of the AE signal, which is typically between 100 kHz and 1 MHz [86,87]. The microcontroller clock should be synchronized at least at 1 μs accuracy to achieve an adequate sampling rate between 1 and 10 MHz, which requires powerful hardware with high power consumption [88]. In addition, the available wireless communications protocols cannot handle this massive stream of data in real time. For instance, acquiring an AE signal at a sampling rate of 5 MHz using a 16-bit analog-to-digital converter (ADC) requires a communication protocol with practical data transmission rate of 80 Mbps, which is a cumbersome task using the available wireless communication protocols. Another limitation for embedding the AE sensor in the TCM sensor node is the size of the commercially available signal conditioners, which may be replaced by a miniature electronic circuit tailored for TCM systems [89]. Available commercial AE wireless monitoring systems are limited to off-line data transmission with a low-frequency range [90]. Additionally, the space and weight required to accommodate such systems impede the integration of this sensor on a rotating tool.

**Figure 5:**
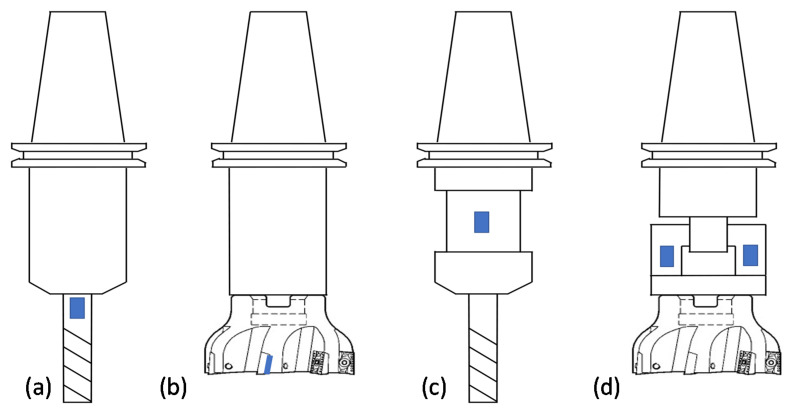
Various concepts of thin film placements as indicated by the blue spots: (**a**) on the tool [93,94], (**b**) under the insert [102], (**c**) on a reduced diameter of the tool holder [106], and (**d**) on an integrated flexible body [91].

To overcome the limited practicality of commercial dynamometers, tool-embedded thin-film force sensors have been proposed within the wireless tool-embedded sensor node concept [91]. Numerous embedded sensors have been used to measure cutting forces and torque, including strain gauges [84,92], piezoelectric polyvinylidene fluoride (PVDF) [93,94,95], semi-conductive strain gauge [96,97], fiber Bragg grating [98], surface acoustic wave [99], capacitive sensor [100], and piezoresistive microelectromechanical systems (MEMS) [91,101]. Based on the reviewed literature, strain gauge and PVDF sensors are the mainstream for detecting cutting forces. However, the PVDF sensor possesses unique characteristics, such as a broad bandwidth with resonance over 10 MHz, high strain sensitivity, high dynamic range [93,94,102]. However, PVDF sensors suffer from charge leaks and are not proper for measuring static forces [103]. Mounting force sensors on the tool holder provides accurate torque measurement [104,105]. For accurate calculation of the cutting power, a gyroscope sensor can be added to the tool holder to monitor the changes in the spindle speed [58]. The gyroscope should have a wide measurement range to be applicable for high-speed milling operations. Several approaches have been proposed for force sensor integration on rotating tools to accommodate differences in the available measuring techniques. Thin force films have been bonded on the tool [93,94], under the inserts [102], on a reduced diameter of the tool holder [106], or on an integrated flexible body [91], as shown in Figure 5. Usually, modifications of the tool holder are made to increase the measurement sensitivity [91]. The desired tool holder modifications should have a simple structure and preserve the system stiffness and the tool compliance with minimum interference with the working space [82,100]. Mounting the film sensors behind the cutting inserts or on the tool directly can comply with most of these requirements. However, the sensors can be deteriorated by the elevated temperature at the cutting zone when milling difficult-to-cut material. In terms of the system stiffness, integrating a flexible body into the system degrades the stiffness to a great extent.

Several wireless sensor nodes have been integrated with multiple sensors, as provided in Table 1, to increase the reliability of the TCM system in the high dynamic environment of the milling process. Xie et al. embedded capacitive sensors and a one-axis MEMS accelerometer into a modified tool holder to measure the triaxial cutting forces, torque, and cutting vibration [79]. A safe operating range of the spindle speed up to 4600 rpm was defined for a tool with two inserts based on the reduction in the system stiffness and the achieved sampling rate. However, the system was tested in the milling of a steel workpiece at a spindle speed up to 2200 rpm only. The wireless sensor node developed by Rizal et al. can monitor up to six variables, including triaxial forces, torque, axial vibration, and tooltip temperature [84,107]. The force sensing element consists of 36 strain gauges that were mounted on a flexible element inside the tool holder. Based on the achieved sampling rate of 5000 Hz of the used telemetry system and the tool holder stiffness, the wireless sensor node can work safely and without distorting the data at spindle speeds up to 5000 rpm for one insert cutter. The proposed wireless sensor node was relatively large and intrusive to the operating field. The industrial wireless sensor nodes on the tool holder are very rare and can measure certain quantities without decision-making systems regarding the tool state. Recently, Pro-Micron has developed a wireless sensor node (SPIKE), which is capable of measuring the torque and two bending moments at a sampling rate of 2500 Hz, as shown in Table 1 [108]. This system has been used to collect cutting force data to monitor the tool wear in [109], chatter [110], and surface roughness in [80]. Another model available in the market has been developed by Schunk GmbH that can measure only the cutting vibrations using a MEMS accelerometer [111]. This sensor node was employed in a TCM system to monitor the cutting tool edge chipping events in the milling process [112]. In terms of the system stiffness and the quantities measured, the developed designs are still limited, and further improvements are necessary.

#### 2.2.2. Data Transmission and Power Management

The common wireless technologies, utilized to transmit data to the host computer in TCM systems, include Wi-Fi [78,79,106,113,114,115,116], Bluetooth [82,95,117,118], and ZigBee [91,93,119,120]. Table 2 provides the specifications of the available wireless communication protocols used in the TCM systems [121,122]. It is worth noting that the typical data speed is much lower than the theoretical one because of, for instance, the packet overhead and delay between packets [123,124]. Based on the presented specifications, Wi-Fi networks have the potential to be strong competitors of other wireless communication technologies for remote and real-time TCM, considering their relatively low latency and high transmission rate but with high power consumption. As shown in Table 1, a maximum sampling rate of 40,000 Hz/channel has been achieved using Wi-Fi networks in [20], which is much lower than their typical transmission rates due to the associated latency and the limited capabilities of the used microcontroller [78,106]. It is recommended to set the sampling rate 5–10 times the maximum frequency of the detected signal to avoid the signal distortion [88,91], which limits the operating range of the spindle speed reported in the literature. Other wireless protocols used for TCM data transmission (Table 1), but with a total sampling rate lower than Wi-Fi protocols due to their limited theoretical transmission capacity, are presented in Table 2. Other limiting factors that control the sampling rate are the speed of the microprocessor and the wired transmission protocols between the analog–digital converter (ADC) and the microcontroller. Therefore, the maximum transmission capacity of wireless networks is commonly not gained in most of the previous work [79,80,106,118]. The high sampling rate achieved in [20] (Table 1) can be attributed to the utilization of the Serial Peripheral Interface SPI transmission protocol, which achieves high speeds compared to the Universal Asynchronous Receiver/Transmitter UART and the Inter-Integrated Circuit I^2^C protocols used in other designs.

Rechargeable batteries are widely used as a power source for wireless sensor nodes. The wireless module and the microcontroller are the dominant power consumers compared to the sensing unit, which usually has the least power consumption at levels of milliwatt [126]. The power consumption of the microcontroller heavily depends on the instructions processed per second. The most critical characteristics of recharged batteries for a TCM sensor node are energy density, fast-charge time in hours, charge/discharge cycle, cell voltage/voltage stability, size, self-discharge rate, and cost. Table 3 compares some of the characteristics of the most common types of batteries that include lead-acid, nickel–cadmium, nickel–metal hydride, and lithium-ion [127]. Each battery type has several advantages and drawbacks when used to power the wireless TCM sensor node. Since no battery technology currently exists that satisfies all these requirements, a trade-off must be made. Lithium-based batteries are the most sophisticated technology and are commonly utilized, as they have high energy density and moderate self-discharge rates compared to other types [78,79,106,119]. However, the voltage stability curve is steep during the discharge compared to other batteries. TCM tool-embedded sensor nodes reported in the literature use DC-DC conversion circuits to power all the node components at different voltage levels using a single battery [78,79,106,119]. It should be noted that batteries represent dead weights that need to be balanced due to the high revolution of the cutting tool, and any error might deteriorate the quality of the machining process and the acquired signals.

To reduce the needed battery sizes and prolong its cycle life, energy harvesting techniques have been utilized to continuously charge the utilized battery based on mechanical vibration energy [128,129,130], electromagnetic energy [120], or utilizing an inductive power transmission system [84,107]. However, the application of such systems is restricted. In the turning and milling operations, the approach offered by Ostasevicius et al. [128,129] to harvest the mechanical vibration energy using a piezoelectric energy harvester was restricted by the narrowness of the frequency bandwidth. Another solution proposed by Chung et al. [120] depends on attaching four magnets on the rotating spindle on the milling machine to induce a current by the coil around the tool holder. Such a system requires at least a speed of 1650 rpm to generate enough energy and it interferes with the working zone. A very relevant approach with the same degree of intrusiveness has been proposed by Rizel et al. [84,107], who used a telemetry condition monitoring system to transfer the energy and data using two inductive near-field coils. Such sources of electromagnetic energy are not favorable for the signal quality, and they require special wiring between sensors and microcontroller [126]. Thus, practical design requirements for designing robust TCM sensor nodes for industrial application necessitate optimized solutions for the power supply and the utilized wireless transmission protocols.

## 3. Signal Pre-Processing

Signal pre-processing is typically needed and executed by a sensor-specific conditioner before or after signal digitalization due to sensor characteristics and the interference caused by mechanical, electrical, and ambient disturbances. The common signal conditioning approaches adopted in the signal pre-processing stage are:

- Amplification: At an early step, the signal is typically amplified due to the low-level output signal of the used sensor, which increases the signal-to-noise ratio and reduces the unwanted interference. The maximum voltage range of the signal should meet the maximum input range condition of the analog–digital converter to achieve the best level of accuracy [17].
- Sampling: After amplifying the signal, the acquired signal should be sampled at a sampling rate more than two times the highest frequency of interest seen in the signal according to the Nyquist–Shannon sampling theorem [88]. In practice, the sampling rate should be 5–10 times the highest frequency of interest for better representation of process variables [20].
- Filtering: Digital or analog filtering is used to exclude the undesired signal frequencies while preserving the correlation between sensor data and process variables, such as studying the cutting force signals at the tool-pass frequency [84]. Filtering is also commonly used to avoid aliasing from high frequency signals, which can be accomplished by attenuating signals above the Nyquist frequency with an anti-aliasing filter. Anti-aliasing filters are appropriate for vibration signals since accelerometer readings are typically evaluated in the frequency domain [88]. In general, the obtained signal can be filtered using high-pass, low-pass, or band-pass to exclude undesired signal frequency components.
- Segmentation: As an optional technique for pre-processing sensor data, segments of the signal are extracted when the tool is engaged with the workpiece material as only these segments include information about the tool condition [8,131]. The most basic and widely used technique of signal segmentation is the detection of a signal value surpassing a predefined threshold in a user-defined time window [8,132,133]. An experimental definition of the threshold value is required because it is determined as part of the maximum signal value. Another segmentation approach can be implemented per tool rotation to produce repeating patterns of the extracted segments [22], where an overlapping time moving frame was applied to avoid disruption of data continuity.

## 4. Signal Processing Techniques

In machining processes, the acquired signals are nonlinear and nonstationary, as well as noisy [134]. Moreover, data are collected continuously at an ever-increasing size with extremely high dimensions, which requires massive storage and computational resources. Retrieving useful and understandable information for the decision-making stage becomes a great challenge [135]. Therefore, representative features are constructed during the signal processing stage as a compact and informative representation for the monitored variables. Incorporating all of the constructed features increases the classification problem dimensionality, with the possibility of including potentially irrelevant, noisy, or redundant features [136]. This can be tackled by implementing dimensionality reduction techniques to select the most informative features to be employed in the decision-making algorithm. In the next subsection, the feature construction approach in the three domains of the time, frequency, and time–frequency domains is discussed, followed by an analysis of the-state of-the-art of dimensionality reduction techniques in Section 4.2.

### 4.1. Features Construction

One of the most crucial stages in TCM systems is feature construction, which determines the success of any classification model [137]. Physical and statistical features that express the input data characteristics are usually constructed in the signal processing stage and are optimized during the dimensionality reduction stage [138]. Most of the monitored variable characteristics can be expressed through extracting representative features in the time, frequency, and time–frequency domains.

Time domain features are the most common and simplest features in terms of extraction and required computations. The most common time-domain statistical features are the average, maximum/minimum, root-mean-square, and peak-to-peak amplitude of the signal. In addition, the probabilistic distribution of acquired data are usually represented through extracting the variance, crest factor, skewness, and kurtosis [139,140]. Moreover, coefficients of time series modeling, such as auto regressive (AR), moving average (MA), and auto-regressive moving average (ARMA), were utilized for TCM [17,25]. Time domain features are commonly used with features from other domains as they are vulnerable to noise and do not provide information about signal frequencies [25,141].

Frequency domain features are constructed by transforming time-series signals into the frequency domain to evaluate the dominant frequency component. The fast Fourier transform (FFT) or its enhanced variants, the discrete Fourier transform (DFT), and discrete cosine transform (DCT) have been commonly used and reported in the literature [142,143,144]. Extracted features include the peak frequency, peak amplitude, spectral crest factor, as well as the mean, variance, skewness, and kurtosis of the band power. The FFT averages the signal frequency contents over the signal time with fixed resolution over the whole frequency spectrum, which makes it inappropriate for the nonstationary signals acquired in milling operations.

Time–frequency domain features can evaluate the signal localization in both time and frequency domains. This domain has attracted considerable attention for TCM systems compared to the aforementioned domains [2,18,25]. Time–frequency representation of the acquired data is constructed using the continuous wavelet transform (CWT), discrete wavelet transform (DWT), wavelet packet transform (WPT), short-time Fourier transform (STFT), or empirical mode decomposition (EMD) algorithms [29,34,145,146]. Extracted features include the average energy of wavelet coefficients and their wavelet domain statistics (RMS, mean, and variance, etc.) [147]. The CWT is computationally expensive and contains plenty of redundant information compared to the DWT [25]. On the other hand, the frequency domain sampling is fixed in the DWT or the WPT, which lead to low resolution, frequency aliasing and insufficient shift-invariance that cause wavelet distortion [2,148]. These shortcomings can be alleviated or avoided by using the tunable Q-factor wavelet transform (TQWT) technique, which is an overcomplete DWT variant [149]. A sparse wavelet energy feature, constructed using the TQWT, showed high-resolution concentrated energy that improved the failure detection of a faulty rolling bearing compared to DWT- and WPT-based features [150]. Based on the surveyed literature, the TQWT has not been applied yet in TCM systems.

One of the most effective methods for time–frequency domain analysis is the empirical mode decomposition EMD that was developed explicitly for nonlinear nonstationary signals using an adaptive data-driven approach [151,152,153]. EMD can adaptively decompose the input signal into a collection of intrinsic mode functions (IMFs) via a signal sifting process, resulting in meaningful instantaneous frequency estimations [154]. However, the noisy and intermittent nature of the acquired data in machining operations can deteriorate the analysis quality by producing mode mixing (a single IMF contains different scales) and mode splitting (the existence of one scale in one or two IMFs) [155]. Newly developed EMD variants have been developed to address mode mixing, such as the ensemble empirical mode decomposition (EEMD) [156], the complementary EEMD [157], the noise assisted multivariate EMD (NA-MEMD) [158], the complete EEMD [159], the partly EEMD [160], and the fast multivariate EMD (FMEMD) [161]. Following the same treatment of the input signal, iterative filtering techniques have been developed to iteratively decompose the input signal using moving average computation, which can guarantee its stability and convergence [162]. This guarantees the elimination of the mode mixing, but alleviating mode splitting requires experimental tuning of the stopping criterion of the sifting process [163]. Iterative filtering techniques include the fast iterative filtering [164] and the adaptive local iterative filtering methods [165]. Although the capabilities of these techniques have enabled their use in a wide variety of applications [163], their use in TCM has not emerged yet [166].

### 4.2. Dimensionality Reduction

The values of constructed features change as a result of variation in cutting conditions, cutting tools and workpiece materials, the type and units of various signals and features, as well as the deterioration of the cutting tool health, among others [167]. They are also sensitive to different sensor sensitivities and performance. Therefore, the ability to construct generalized tool condition descriptive features has been limited, resulting in TCM systems that lack certainty and generalization [168]. It is crucial to isolate all causes of candidate features variation while retaining variations due to the tool health state, and minimizing the time required for the learning process [22,167]. To address these issues, feature normalization techniques have been suggested to provide features highly sensitive to the tool health state using mean, standard deviation, or extreme values of candidate features or using empirical formulas for cutting conditions [167,169,170,171]. Another approach has been adopted by eliminating features that are highly dependent on cutting conditions and less sensitive to the tool health state using ANOVA and f-test [22].

High-dimensional data are another issue in TCM systems that results from constructing features in multiple domains, which increases the computational cost in the training stage and degrades the classifier’s accuracy if insignificant noisy features are included [2]. A model trained on a large number of features becomes excessively dependent on the data, resulting in overfitting and poor performance on the new dataset [172]. Therefore, dimensional reduction methods are adopted in TCM systems through mapping the high-dimension data to a lower dimension space by selecting and extracting the most discriminative and dominating features out of the initial global feature set. During the offline model training, a limited number of features that are highly indicative of the tool state should be carefully identified to develop an accurate and computationally efficient TCM system during the online implementation stage [38]. In multi-sensor based TCM approaches, numerous advantages can be gained by employing dimensionality reduction techniques on the full feature set: (1) significant reduction in the computational time and the needed data storage space; (2) more efficient and accurate AI classifiers can be developed by eliminating noisy and misleading features; and (3) the ability to evaluate and visualize patterns in data and outliers, leading to a better understanding of the classification problem [38,135,137]. Dimensionality reduction can be implemented through two approaches, namely feature selection and/or feature transformation.

#### 4.2.1. Feature Selection

**Figure 6:**
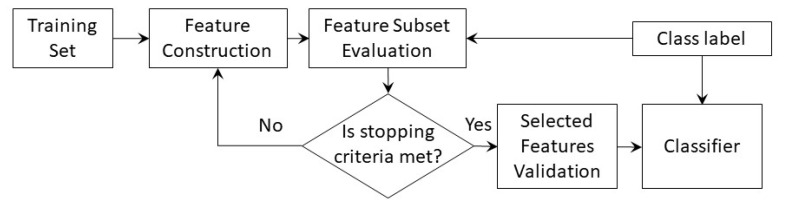
Filter techniques framework used for feature selection.

**Figure 7:**
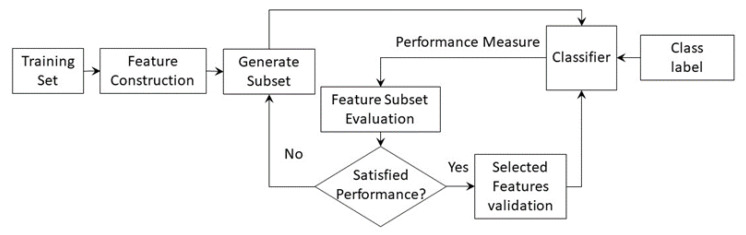
Wrapper technique framework used for feature selection.

**Figure 8:**
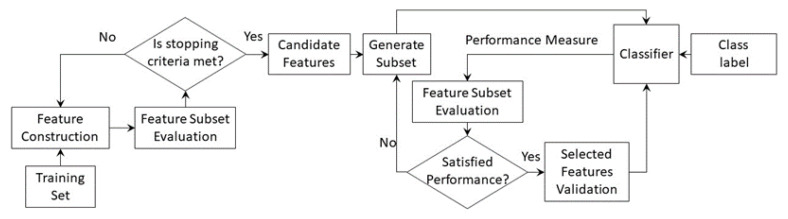
Hybrid technique framework used for feature selection.

Subset feature selection techniques are used to select the most discriminative features of the tool health state to minimize the computational effort and to increase the accuracy of the classification model. No relevant information can be lost during the feature selection process. Typically, conventional feature selection techniques rank the extracted features based on their sensitivity to tool condition and then choose the top-ranked features. The feature selection techniques can be categorized as follows [173]:

- Filter techniques are open-loop computational methods that only consider the relationship between features and class label without involving the subsequent tool wear classification model, as shown in Figure 6. They evaluate the usefulness of features subsets based on their intrinsic properties using evaluation measures, such as dependency, consistency, or information, to eliminate low-ranking features [171,174,175]. The ranking measure is determined using statistical measures, such as Pearson’s correlation coefficient, the coefficient of determination, minimum redundancy maximum relevance (mRMR), or analysis of variance ANOVA [171,174,176,177,178]. A detailed discussion on various performance measures is available in [179]. Filter techniques have relatively low computational cost and high scalability to large feature datasets. Their major drawback is the non-involvement of the decision-making algorithm, which makes its accuracy data-dependent.
- Wrapper techniques are closed-loop techniques, in which the tool wear model is used for selecting the most discriminative features by minimizing the misclassification error of the model, as shown in Figure 7 [180]. Several models have been developed by training the classification algorithm using different subsets of features in order to define the optimum subset with minimum classification error. In terms of classification accuracy, wrapper techniques outperform filter techniques. Additionally, they consider the dependencies among selected features [137]. However, the primary downsides of this technique are the expensive computational effort to achieve convergence, and being more prone to overfitting, compared to filter techniques. Feature subsets are usually generated using heuristic or random search strategies [181,182]. Forward and backward sequential selection methods are used by sequentially adding or removing one feature at a time, respectively, until a local maximum accuracy is achieved [183]. Because both methods ignore the inter-dependency of features, sequential floating forward selection (SFFS) and sequential floating backward selection (SFBS) were developed [184]. The genetic algorithm (GA) [185] and the ant colony algorithm [186] are among the most representative methods of random search strategies that have been used to optimize the constructed features and to select features with a high correlation with the tool health state [187].
- Hybrid techniques are the result of merging a wrapper technique and a filter technique to inherit the complementary strengths of both models, as shown in Figure 8 [137]. For model-independent techniques (wrapper and embedded techniques), the size of the candidate feature dataset should be kept modest; otherwise, a significant amount of training data will be required [188]. Therefore, the filter technique using the mRMR is applied to remove irrelevant features and to alleviate redundancy among features. It has been successfully applied to reduce the feature dataset size by 1000-fold in grinding operations [177]. This preselection stage is followed by applying the wrapper technique through assessing the model performance using the coefficient of determination and root-mean-square error (RMSE).
- Embedded techniques are built-in feature selection techniques, in which the feature selection and the model training processes are merged [181]. During the model training, a scaling factor is assigned to each candidate feature, and it is optimized to reflect its relative relevance [189]. Embedded techniques have the merits of the filter and wrapper techniques. They are computationally efficient while maintaining a classification accuracy comparable to wrapper techniques owing to omitting the repeated execution and evaluation of each feature subset by the learning algorithm. However, they are classifier dependent, and their performance might degrade if the initially constructed dataset has several irrelevant features [137]. A gradient boosting decision tree (GBDT) is an example of embedded techniques in TCM systems that were used to optimize the size of the initially constructed feature dataset from 198 features to 40 discriminative features in tool wear monitoring application [190]. Abubakr et al. used a random forest (RF) classifier, in which out-of-bag (OOB) samples are utilized to reduce the constructed feature pool from 152 to 15 features while maintaining a high level of classification accuracy [139].

#### 4.2.2. Feature Transformation

The feature transformation methods implement algebraic feature transformation to the input feature set, according to some optimization criteria, to develop a low-dimensional representation to reduce the required computational resources [191]. The output of the process is a dataset of artificial features that retains the characteristics of the input feature set without losing information. In contrast to feature selection algorithms, the size of the constructed features can be reduced with minimal sacrifice of the information stored in the initial feature dataset [192]. However, the original characteristics in the transformed features are inexplicable, and information about the contribution of each original feature is frequently lost [193]. A wide variety of feature transformation algorithms has been developed, but the selection of a certain algorithm is highly dependent on the characteristics, quality, and quantity of the data [135].

Algorithms for feature transformation can be classified as linear or nonlinear algorithms [135]. Linear algorithms transform a high-dimensional feature space into a lower dimensional feature space with a linear combination of the original dimensions. Principal component analysis (PCA) [61,194,195], singular value decomposition (SVD) [196], linear discriminant analysis (LDA) [197], Fisher discriminant analysis (FDA) [198], Fisher discriminant ratio (FDR) [199], factor analysis (FA) [200], and independent component analysis (ICA) [201] are examples of linear feature transformation algorithms. On the other hand, nonlinear algorithms, such as kernel PCA (KPCA) [202,203], probabilistic kernel FA (PKFA) [200], kernel Fisher discriminant analysis FDA (KFDA) [204], and isometric mapping (ISOMAP) [205], nonlinearly transform a high-dimensional feature space into a lower space. Feature transformation algorithms can be categorized according to the need for pre-existing class labels into supervised algorithms, such as LDA and ICA, and unsupervised algorithms, such as PCA, KPCA, SVD, and ISOMAP.

PCA is a popular linear unsupervised feature transformation algorithm that orthogonally projects features into a synthetic feature domain, based on their variances in which features with low variance are disregarded [61,179,206]. Its objective is to extract critical information from the data and represent it as a collection of new orthogonal variables (principal components). Caggiono et al. have conducted two-stage dimensionality reduction approach through applying Spearman’s rank order correlation (filter technique), followed by PCA to represent the tool wear level using only two features with high accuracy [206]. The final extracted features are linear combinations of the original feature constructed from multiple signals, which preserves the sensor fusion approach with minimum computational cost. PCA helps in the removal of noise from datasets and makes it easier to explore and visualize a low-dimension dataset. However, it was primarily employed to extract linear features, resulting in the loss of valuable nonlinear features. KPCA was proposed to investigate the nonlinear relationship between variables using the kernel function. KPCA is an unsupervised feature transformation algorithm that can handle non-Gaussian, non-linear, and nonstationary signals [202]. Lee et al. [68] demonstrated that the accuracy of KPCA is superior to the accuracy of PCA, decision tree (DT), K-nearest neighbors (KNN), Naive-Bayes classifier (NBC), and quadratic discriminant analysis (QDA). The linear factor analysis FA and its nonlinear variant PKFA are Gaussian latent variable algorithms. In an investigation conducted by Wang et al., PKFA was found to outperform PCA, KPCA, and FA, when used in conjunction with a support vector regression (SVR) model in TCM [200]. Isometric mapping ISOMAP is a nonlinear unsupervised algorithm that retains the distance between points and considers the neighboring data distribution, unlike the PCA algorithm. ISOMAP has been integrated with expectation-maximization PCA (EM-PCA) to reduce the dimensionality of the constructed features on two stages to create a single health indicator per signal that was used as an input for a SVR model to predict cutting tool wear level [205]. LDA can be used as either a supervised linear feature transformation algorithm or a linear classifier [22,197]. In feature transformation applications, LDA determines a new feature space by projecting the input features with the objective of maximizing the separability of classes [197]. For non-Gaussian and small sample size data, LDA is ineffective. Therefore, subclass discriminant analysis (SDA) [148] and mixture subclass discriminant analysis (MSDA) [207] have been proposed to overcome these issues [208]. Because most of the transformation algorithms create new features without interpretable physical meaning, FDA can be used to keep the physical meaning of the constructed features, which assists in promoting the data interpretability based on the process physics during the system performance tuning [198].

## 5. Decision Making for Tool Wear Monitoring

Classifier-based machine learning algorithms have been extensively applied to support the decision-making stage, particularly to monitor the progressive tool wear [15]. Promising results for the prediction of the tool health state have been reported to optimize the service life of the cutting tool by preventing early replacements and limiting scraps by preventing part damage [6,62]. Popular machine learning classifiers for tool wear monitoring include artificial neural network (ANN) [209], SVM [210], Bayesian networks [211], hidden Markov model (HMM) [212], DT [21], KNN [20], Gaussian process regression (GPR) [213], and fuzzy logic [36]. These algorithms are commonly fed by hand-crafted features and come with their own set of advantages and limitations, as has been extensively discussed in [15,16,25]. Although ANN has been widely used in TCM systems due to its adaptability and robustness, it has several drawbacks, including slow convergence, local minima, and the need to tune multiple biases and weights [214].

In addition to the conventional machine learning techniques, researchers have employed adaptive neuro-fuzzy inference system (ANFIS) [215], relevance vector machine (RVM) [216], and random forest (RF) [217] in TCM systems to monitor tool wear. In these studies, fewer than ten indicative features were usually extracted by the system developer and fed as an input to the classifier. The feature selection procedure is not only time-consuming and requires the expertise of feature engineering, but the sensitivity of selected features may also be lowered if the conditions, to which the model is tuned, are changed. Additionally, these models are almost shallow with limited generalization ability, which can be attributed to the limited capacity to simulate complicated nonlinear behavior of machining operations [218]. To increase the robustness and the prediction accuracy of the TCM system, the fusion of data-driven models has been proposed [71]. The final decision is determined using a voting function over the different classifier outputs. Kannatey-Asibu et al. improved the classification rate by 12% using a penalty-weighted voting factor for four classifiers, which came at the cost of the computational effort [71]. Another hybrid approach has been followed by combining a physical model with a data-driven model to improve the predictions in [9,219]. Despite the remarkable decrease in the prediction error by up to 50%, the computational cost increased, and the hybrid approach was tested at very limited cutting conditions.

Recently developed advanced deep learning methods, such as convolutional neural network (CNN) [220], recurrent neural network (RNN) [221], deep belief network (DBN) [222], and sparse autoencoder (SAE) [223], have been employed in TCM systems. Deep learning methods can implicitly extract representative features themselves; however, a surplus of training data is needed [92,224]. Without involving feature engineering and the needed expertise, an online tool wear model based on CNN [222] has achieved a classification accuracy of around 78%, which can be improved further by employing indicative features from the acquired data. The DBN has been compared to SVR and ANN and showed superior prediction stability when used to monitor tool wear in milling operations using force, vibrations, and AE data [225]. Four gradual wear stages of the cutting tool in the milling operation in two different manufacturing environments were identified using an unsupervised model based on SAE in [226]. However, a threshold value is needed at the end of the model to scale the mean error sequence of the SAE to reflect the cutting wear trend. To decrease the learning effort, Hassan et al. [48] trained a unified LSTM-RNN architecture using a biased dataset taken from a single cutting condition combination, resulting in a 75% decrease in learning effort, when compared to the previous work, and processing time within 1 ms. The key advantage of LSTM is the ability to capture long-term dependencies in the monitored signal, in contrast to the previously mentioned methods. The model was only able to categorize the state of the tool health into either fresh or worn tools. Recently, a hybrid model based on wavelet scattering and CNN was used to select informative features for an LDA wear classification model for a wide range of cutting conditions and different materials [227]. The tool health condition was classified into three stages, namely, fresh, usable, and worn status, with less learning effort and higher prediction accuracy. Although the industrial requirements were met by both works in [48,227] in terms of learning effort, accuracy, and generalization, these TCM systems lack providing a warning stage for the tool condition to allow a tool change before the end of the tool life. Furthermore, the real challenge is to maximize the tool remaining life so that it can finish at least an ongoing machined feature before replacing the tool. The automation of such an industrial approach requires synchronization between the TCM system, machine controller, and predefined G-code sections, which is not a trivial task.

To reduce the learning effort, deep transfer learning approach has been proposed recently in TCM applications, where a classification model that was developed for a specific application is reused as a starting point to develop a new model for another application [225,228,229,230]. The transferability of a pre-trained deep network can be achieved by either weight update, feature transfer learning, or weight transfer [225]. Image classification deep networks have been utilized for TCM by fine-tuning them using tool failure data [228,230]. TCM models developed for a certain tool can also be utilized through transfer learning to monitor unlearned tools with different geometric and material features while minimizing the development efforts and lead time. For turning operations, Sun et al. showed that the transfer learning capability can increase the prediction accuracy of tool wear level, compared to developing a model from scratch, when the same training effort is utilized [225].

AI model interpretation increases trust in the rendered decisions as they can be logically assessed. Several conventional classification models, such as linear or tree-based models, are easy to interpret and, hence, can be physically linked to the cutting process. However, this comes at the expense of their biased performance, which reduces the model accuracy. Deep machine learning algorithms overcome this performance limitation but are difficult to interpret and, hence, are not the recommended approach in industrial applications, where a false rendered decision could have a high cost impact on the industrial facility. A desirable solution would use an interpretable model with low-variant key features that are directly linked to tool wear and insensitive to the cutting conditions.

Low computational and decision-rendering times are essential for successful real-time implementation of TCM systems. Hassan et al. benchmarked the computational time needed by different machine learning algorithms, including SVM, LDA, ANN, and KNN using the same set of features to define the tool condition [231]. The KNN algorithm has utilized the maximum computational effort to render a decision, with an average computational time of 115 µs. This shows the practicality of applying AI-based decision-making algorithms in real-time TCM applications. However, the time needed for signal acquisition and conditioning and feature extraction should also be considered.

Despite the utilization of the most advanced conventional and deep learning algorithms, no comprehensive, reliable, and friendly solution to monitor tool wear in real-time has been found yet in the open literature to satisfy the industrial environment requirements. They are commonly trained and validated on a single machine tool, with a single cutting tool-workpiece combination, and under a limited set of cutting conditions [22,232], as investigated in [34,35,146,233]. This led to the absence of generalized discriminative features that are capable of describing the tool condition under a variety of processes and cutting conditions [4]. In milling operations, investigating the correlation between the tool health deterioration and cutting parameters, such as the chip segmentation characteristics, the rubbing force, the tooth-pass frequency, and the direction of the resultant cutting force, may help in discovering novel variables or features. Such investigations may have the potential of increasing the practicality of TCM systems to meet the industrial requirements.

## 6. Integrated Tool Breakage/Chipping Monitoring and Decision Making

High stresses and excessive heat at the tool cutting edge are the common causes for sudden tool failures in the form of chipping or/and breakage, particularly when machining a difficult-to-cut material [62,234]. Early detection of the tool chipping/breakage would protect the machined part, which satisfies the increased demands for cost-effective and high-productivity machining operations [65]. It would also ensure better product quality by safeguarding the machine tool components and workpiece. Compared to wear monitoring studies, few investigations have been conducted to monitor tool chipping and/or breakage in milling operations. Using a tool holder sensor node equipped with a single-axis MEMS accelerometer [111], the tool chipping was addressed by monitoring the change in the cutting conditions just after the chipping occurred [112]. Different sizes of tool chipping were artificially created on the tool inserts and detected using a fast algorithm based on extra trees classifiers (ETC). By mounting an accelerometer at the fore bearing of the spindle, Mou et al. [234] detected the gradual tool microchipping when milling a Ti6Al4V workpiece. A moving average root-mean-square (MARMS) and a peak power spectral density (PPSD) estimate based on the Yule–Walker method were utilized as indicators. In addition, to avoid the false alarms caused by the continuous change in the cutting conditions, the signal segments of interest were extracted by establishing a communication between the proposed approach and the numerical control (NC) blocks. During the end-milling of Inconel, Kang et al. [235] monitored the time between two consecutive vibration signal peaks (peak period) to detect the radical change in the tool geometry due to chipping. A threshold value based on the experimental results was set to define the chipping events after masking out the tool runout and its consequences on the peak periods of the unworn tool. The cutting forces were also used to detect the tool chipping after being estimated from tool bending measurements using an eddy current sensor mounted on the machine spindle to avoid the intrusiveness of the table dynamometer to the cutting zone [236]. The estimated cutting forces were fed into a mechanical model to estimate the change in the tool eccentricity caused by the tool chipping. All the aforementioned research can detect tool chipping/breakage only *after* it has taken place. They cannot predict and prevent the tool failure, which jeopardizes the economics of the machining operations. Duo et al. collected several external and machine internal signals to predict the tool breakage due to excessive tool wear in drilling operations [65]. They concluded that cutting force signals acquired by external sensors or internal signals expressing cutting forces are accurate for tool breakage prevention.

Very few studies have been concerned with providing an online prediction and prevention of sudden tool chipping or breakage through monitoring the unstable crack propagation in the pre-failure stage, which has been presented by Hassan et al. [41,237]. The proposed failure prediction system in [41] is based on signal conditioning the bursts in the RMS values of the AE signal due to tool cracks caused by the excessive mechanical stresses on the tool tip in the aluminum intermittent operations. Compared to vibration signals, AE is well recognized for its ability to detect the deformation or fracture of the materials under stress by monitoring the transient elastic wave that emits from generating new surfaces during unstable crack propagation [238,239]. To handle the nonstationary and nonlinear RMS signal of the AE, a Teager–Kaiser energy operator-Hilbert–Huang transform (TKEO-HHT) processing approach was developed. This approach correctly predicted tool chipping within a window processing time of 2 ms, which allowed sufficient time to stop the machine before tool failure [41]. The approach was only demonstrated during cutting high thermal conductivity work materials, such as aluminum alloys, where the thermal effect on the tool failure behavior and acquired signals is insignificant. The quality of the detected signal might be affected by the signal transmission path between the cutting zone and the AE sensor when a different machine is used. Therefore, employing a sensor node on the tool holder with an integrated AE sensor may be a good candidate solution to increase the versatility of the proposed approach. Additionally, a learning function for correlating the extracted features in the prefailure phase with the chip size is needed to automate the threshold definition process.

## 7. Conclusions and Future Research Avenues

Numerous TCM systems have been developed to detect tool wear, chipping, and breakage in laboratories around the world. Various data acquisition, processing, and decision-making AI techniques have been proposed in an attempt to develop an industry-oriented TCM system. Despite the fact that TCM research has made significant progress, components of the TCM system have several shortcomings that require further investigation. The following are the conclusions and future recommendations for an industry oriented TCM system:

- Data acquisition: Until recently, previous TCM research has adopted a conventional approach, in which the sensors are mounted on the machine spindle or the workpiece. In this approach, vibration and acoustic emission AE sensors were preferred in industrial TCM applications, since monitoring cutting forces using a table dynamometer is impractical due to the high intrusiveness and investment cost. Although the cutting tool temperature is correlated to tool wear in the milling operations of difficult-to-cut materials, such as Ti6Al4V alloy, it is not widely monitored and used as an indicator of tool wear. The quality of the vibration and AE signals are impacted by the long signal transmission path and multiple reflective surfaces, particularly when they are mounted on the machine spindle. This problem can be alleviated by using a universal wireless tool-embedded sensor node in the TCM system. The application of this approach is still limited. Recently, some researchers were able to integrate force, vibration, and temperature sensors into the tool holder to increase the universality of the TCM system. To date, AE has not been integrated yet on a rotating tool for milling operations, due to the complex signal conditioning electronics needed to fit in a confined space on the tool holder. For the conventional multi-sensor approach, optimizing the sensors’ selection and location need to be further investigated, based on the availability and ease of integration of the sensors, as well as the TCM system performance and economics. In the reported commercial and academic designs of TCM tool-embedded sensor nodes, no optimization has been attempted for the selection of the sensors type, proximity, and orientation. Additionally, none of the reported designs have been tailored for machining difficult-to-cut materials, where high cutting forces and concentrated heat can be encountered, causing sensor drift. The sampling rate and the reduced structural stiffness are still the main obstacles for developing a reliable universal wireless sensor node. Furthermore, a comprehensive solution for the power management of the senor node has not been realized yet to reduce the interruptions and/or the intrusiveness to the machining process.
- Feature construction and dimensionality reduction: The TCM research to date has focused on using conventional features constructed from the time, frequency, and time–frequency domains, rather than discovering novel features that correlate to the tool health state, while being independent of the cutting parameters. The compatibility of such features with the most up-to-date tool wear modeling algorithms should be investigated. The time–frequency domain has attracted attention of many researchers in TCM applications, particularly the EMD technique and its variants due to its ability to handle nonlinear nonstationary signals. This technique is, however, susceptible to mode mixing and mode splitting. The fast iterative filtering technique can tackle these issues but its application is still limited in TCM. The generality, adaptability, and computational cost of AI algorithms can be improved by monitoring new variables or discovering new features. Based on the reviewed literature, there has been no detailed investigation of the tool wear impact on the chip segmentation and its consequences on the acquired signals, rubbing force, tooth-pass frequency, and the direction of the resultant cutting force. Such investigation may help to explore a robust feature or variable that decreases the learning effort and increases the generality of the developed model. Due to the great impact of dimensionality reduction on the performance and accuracy of machine learning algorithms, a wide variety of dimensionality reduction strategies have been proposed in recent decades to address the problem of high-dimensional data in TCM applications. The two main adopted techniques are feature selection and feature transformation. However, there is currently no universal strategy for dimensionality reduction that can be applied to all scenarios. Future TCM research should consider developing techniques that are tailored for the nature of acquired sensory data in machining operations to improve the accuracy and robustness of the TCM system.
- Decision making for tool health state monitoring: A remarkable interest has been given to tool wear monitoring with the anticipated increase in utilizing more advanced artificial intelligence AI techniques. Conventional algorithms such as ANNs became more popular for tool wear modeling. Choosing the appropriate algorithm is impacted by the information content and quality of the processed signal. This necessitates extensive research into the efficacy of various signal features and signal processing techniques before implementing the monitoring AI algorithms needed to reduce the learning effort and improve the TCM system generalization. The complex nonlinear and nonstationary nature of machining processes has led to utilizing advanced deep learning algorithms. To overcome their main limitation, few studies have recently been conducted to propose a practical deep learning algorithms with low training efforts to increase the possibility of adopting them for industrial TCM systems. The vast majority of the previous research has focused on exploiting process-feedback signals to identify changes in cutting parameters following tool chipping and/or breakage in machining operations. However, it is crucial to predict and prevent sudden tool failures by chipping and/or breakage *before* it happens. Only one research work has been found that can monitor the unstable crack propagation stage in stationary tools before the occurrence of chipping and/or breakage in intermittent machining operations using an AE sensor by pre-setting a threshold based on experimental results. A fully automated and comprehensive solution for milling operations still needs to be developed. There is also a need for establishing a correlation between the AE bursts and the chipped material, as well as optimizing the location of mounting the AE in the milling machine to reduce the signal transmission path and multiple acoustic wave reflections.

Other issues that require additional developments for the acceptance and implementation of TCM systems by industry include: (a) handling the quantity of data required to effectively train the available data-driven models, (b) any feature selection/transformation and threshold value pre-setting should be handled entirely by the TCM system with minimum intervention from the operator, and (c) optimizing the tool remaining life so that it can complete at least one continuous machined feature before being replaced. This can be achieved by integrating a TCM system with an adaptive control (AC) system, in which the signal behaviors are learnt to manipulate the operating conditions. Such integration is effective and robust but more complex and needs further research.

## Figures and Tables

**Table 1:** Comparison of several wireless TCM tool-embedded sensor nodes.

Author	Forces			Vibrations	Temperature	Wireless Protocol
	Axis ^1^	Design	Sensors	Axis		(Data Rate)
Zhou et al. [20]	-	-	-	*x*, *y*, *z*	-	Wi-Fi (40,000 S/channel)
Luo et al. [102]	*F_x_*, *F_y_*, *F_z_*	Under inserts	PVDF	*-*	-	Wi-Fi (20,000 S/channel)
Xie et al. [79]	*F_x_*, *F_y_*, *F_z_*, *M_z_*	Modified tool holder	Capacitive	*x*	-	Wi-Fi (5000 S/channel)
SPIKE [80,108]	*M_x_*, *M_y_*, *M_z_*	Unknown	Unknown	-	-	Wi-Fi (2500 S/channel)
Wu et al. [106]	*F_z_*, *M_z_*	Modified tool holder	Strain gauge	*-*	-	Wi-Fi (1000 S/channel)
Nguyen et al. [95]	*M_z_*	On the tool holder	PVDF	*-*	-	Bluetooth (13,000 S/channel)
iTENDO [81,111]	*-*	-	-	*x*	-	Bluetooth (9500 S/channel)
Qin et al. [91]	*F_z_*, *M_z_*	Flexible element	MEMS	*x*	-	Zigbee (250 Kbps)
Rizal et al. [84]	*F_x_*, *F_y_*, *F_z_*, *M_z_*	Flexible element	Strain gauge	*z*	√	Telemetry (5000 S/channel)

^1^ *F_x_*, *F_y_*, and *F_z_* are cutting forces and *M_x_*, *M_y_*, and *M_z_* are moments in *x*, *y*, and *z*-directions, respectively.

**Table 2:** Specifications of wireless communication protocols for TCM sensor nodes.

Technology	Data Speed Theoretical (Mb/s)	Data Speed Typical (Mb/s)	Latency (ms)	Range Indoor (m)	Trans. Power (mW)	Sleep Power (mW)	Author
Wi-Fi n/g	75	54	1.5	50	350	300	[20,78,114]
Bluetooth	1–3	0.7–2.1	6	30	-	-	[81]
Bluetooth LE	0.125–2	0.27–1.37	2.5	10	60	8	[125]
Zigbee	0.25	0.15	140	30	72	4	[119,120]

**Table 3:** Characteristics of rechargeable batteries.

Technology	Lead Acid	Nickel–Cadmium	Nickel–Metal Hydride	Lithium-Ion
Energy density (Wh/kg)	35–50	30–60	60–80	80–180
Self-discharge/month	2–8%	5–15%	15–25%	2–10%
Fast-charge time (hour)	8–16	1	2–4	1–4
Charge/discharge cycles	250–1000	1000–50,000	300–600	3000
